# Editorial: Plant diversity: the key to ecosystem resilience in a changing world

**DOI:** 10.3389/fpls.2024.1534119

**Published:** 2025-01-13

**Authors:** Claudia Bita-Nicolae, Praveen Dhyani

**Affiliations:** ^1^ Taxonomy, Ecology & Nature Conservation Department, Institute of Biology Bucharest of the Romanian Academy, Bucharest, Romania; ^2^ Institute for Integrated Natural Sciences, University of Koblenz, Koblenz, Germany

**Keywords:** plant communities, ecosystem stability, abiotic stressors, climatic conditions, biodiversity

In a world shaped by climate fluctuations (Ditlevsen et al., 2002), natural disasters and changing human impacts (Benevolenza and DeRigne, 2019), the richness and diversity of plant species within ecosystems are key factors influencing the resilience of the ecosystem. Plants are fundamental species that, at the base of the trophic pyramid, provide food, shelter and resources to many other species (Christenhusz and Byng, 2016). Our Research Topic, “Plant Diversity: The Key to Ecosystem Resilience in a Changing World”, explored in depth the complex interplay between plant diversity and ecosystem resilience (Wang et al.; Song et al.; de Tomás Marín et al., Zhang et al.; Wei et al.). We revealed the remarkable ways diverse plant communities support productivity (Teng et al., Li et al., Kim et al.), facilitate nutrient cycling and enhance soil stability (Zhou et al., 2024), thereby strengthening an ecosystem’s ability to withstand and recover from a range of disturbances (Gazoulis et al.). These insights contribute significantly to conservation strategies and land management paradigms, guiding conserving and revitalizing ecosystem stability in our planet’s changing global transformations (Li et al.; Kim et al.).

Ecological research showed that diverse plant communities are essential for the stability of ecosystems (Wang et al.; de Tomás Marín et al.). They provide essential functions such as nutrient cycling, pest and disease resistance, habitat provision and support for pollination and reproduction. These multiple benefits enhance an ecosystem’s ability to respond to challenges and sustain its vitality in the face of adversity (Song et al.). In contrast, simplified landscapes with reduced plant diversity are inherently more vulnerable and less able to respond to the changing conditions of our world (Zhou et al., 2024).

Wang et al. investigated how abiotic stressors influence community assemblages in grasslands on the Tibetan Plateau and Mongolian Plateau by examining the distribution of plant traits (height, specific leaf area and leaf dry matter content). The study emphasized that future climate change, including warming and changing rainfall patterns, will affect all communities differently in regions with distinct climatic conditions. At the same time, Song et al. explored the role of plant functional traits in restoring ecosystem functions in karst desertification areas. In this context, de Tomás Marín et al. examined how environmental conditions influence plant community assemblages in sub-Mediterranean ecotones in central Spain. It analyzes functional traits in six plant communities, finding that community type is the main driver of differences in functional structure. Intraspecific trait variability, rather than species turnover, plays a key role in functional changes. The study suggests that ecotones are sensitive to minor environmental changes, leading to changes in plant and functional composition. Teng et al. investigated the net ecosystem productivity of a desert riparian forest over seven years, focusing on how meteorological factors like solar radiation, temperature, humidity, and vapor pressure deficit influence net ecosystem productivity. The findings showed significant daily and seasonal variations in net ecosystem productivity, with a circadian rhythm linked to meteorological conditions. Diurnal temperature and vapor pressure deficit changes were particularly impactful, affecting net ecosystem productivity’s daily amplitude and phase. The study highlights how climate variability influences ecosystem carbon dynamics, offering insights into the potential effects of climate change on arid ecosystems.

There were studies highlighting the importance of biodiversity and ecological processes in different contexts. Li et al. examined how shallow tillage affects species diversity and community dynamics in the Mu Us Desert. It found that shallow tillage increases species and phylogenetic diversity and alters community structure by reducing plant competition and enhancing stochastic processes like dispersal limitation. The study suggests selecting species with high adaptability and low niche overlap for effective ecological restoration. Gazoulis et al. discussed how incorporating non-crop plants, especially weeds, into agroecosystems can improve sustainability and resilience. Properly managing weeds through practices like mowing or grazing can provide valuable ecosystem services. Overcoming challenges related to training and quantifying these services requires research, education, and policy support. On the other hand, Kim et al. focused on the endangered plant *Pedicularis hallaisanensis* in Korea, which has a biennial lifecycle and distinct flowering cohorts. Human-induced habitat changes have reduced its genetic diversity. The study recommends minimizing anthropogenic impacts and including individuals from both flowering cohorts in conservation efforts to preserve genetic diversity. Zhang et al. found that intercropping soybean and maize significantly enhanced root exudates, lowered soil pH, improved nutrient availability (e.g., nitrogen and phosphorus), and increased arbuscular mycorrhizal fungi (AMF) colonization, improving AMF community composition. Metabolomics and sequencing analysis showed that root exudates in this system are closely linked to AMF and soil nutrient dynamics. These findings suggest that increased root exudates in intercropping systems enhance AMF composition, boost soil fertility, and sustain intercropping benefits.

Finally, Wei et al. investigated the effects of plant functional group removal on community biodiversity and niche dynamics in an alpine meadow over 3 and 10 years. Results showed that removing plant functional groups led to changes in species niches. Over time, removing Gramineae and Cyperaceae reduced their numbers, narrowed their niche widths, and decreased niche overlap. The loss of species diversity caused significant changes in the niches of remaining species, with increased negative species associations. Niche differences, driven by resource allocation, were vital in shaping species composition dynamics in the community.

The authors contributing to this research explored how plant diversity influences ecosystem resilience, including its impact on nutrient cycling, productivity and interactions with other species. The authors analyzed how different disturbances, from natural events to human-induced activities, interact with plant diversity to form resilience. Recent advances in ecological modeling, remote sensing technologies, and genetic analysis have allowed further exploration of these dynamics.
